# Hydrogen(H2) treatment for acute erythymatous skin diseases. A report of 4 patients with safety data and a non-controlled feasibility study with H2 concentration measurement on two volunteers

**DOI:** 10.1186/2045-9912-2-14

**Published:** 2012-05-20

**Authors:** Hirohisa Ono, Yoji Nishijima, Naoto Adachi, Masaki Sakamoto, Yohei Kudo, Jun Nakazawa, Kumi Kaneko, Atsunori Nakao

**Affiliations:** 1 Department of Neurosurgery, Nishijima Hospital, Numazu City, Sizuoka, Japan; 2 Department of Surgery, University of Pittsburgh, Pittsburg, USA

## Abstract

**Background:**

We have treated 4 patients of acute erythematous skin diseases with fever and/or pain by H_2_ enriched intravenous fluid. We also added data from two volunteers for assessing the mode of H_2_ delivery to the skin for evaluation of feasibility of H_2_ treatment for this type of skin diseases.

**Methods:**

All of the four patients received intravenous administration of 500 ml of H2 enriched fluid in 30 min for more than 3 days except in one patient for only once. From two volunteers (one for intravenous H2 administration and the other for H2 inhalation), blood samples were withdrawn serially and air samples were collected from a heavy duty plastic bag covering a leg, before, during and after H2 administration. These samples were checked for H2 concentration immediately by gas chromatography. Multiple physiological parameters and blood chemistry data were collected also.

**Results:**

Erythema of these 4 patients and associated symptoms improved significantly after the H2 treatment and did not recur. Administration of H2 did not change physiological parameters and did not cause deterioration of the blood chemistry. The H2 concentration in the blood from the volunteers rapidly increased with H2 inhalation and slowly decreased with cessation of H2 particularly in the venous blood, while H2 concentration of the air from the surface of the leg showed much slower changes even after H2 inhalation was discontinued, at least during the time of sample collection.

**Conclusion:**

An improvement in acute erythemtous skin diseases followed the administration of H2 enriched fluid without compromising the safety. The H2 delivery study of two volunteers suggested initial direct delivery and additional prolonged delivery possibly from a slowly desaturating reservoir in the skin to the surface.

## Introduction

Severe and acute erythematous skin diseases usually require immediate medical attention, particularly when the symptoms involve severe pain and/or fever. Treatment may have to be initiated before spending enough time and effort for investigating real causes of the rush or functional state of the other organs and the steroid agents tend to be the first choice of the treatment. However, the complications from the general use of steroid have been well known and therefore, non-dermatological clinics like ours frequently encounter difficulty in finding quick remedies with minimal side effects. Erythema is reddening of the skin due to inflammatory mechanisms either as primary culprits or secondary features and locally released inflammatory cytokines such as TNF-α, IL-1,8, GM-CSF etc., stimulate phagocytes and inflammatory cells and results in production of ROS (reactive oxygen species)[1,2]. The interaction between the ROS and nitric oxide leads to the formation of peroxynitrite radicals and also by the iron-mediated Fenton reaction, hydroxyl radicals, both of which are highly reactive and destructive to the cell membrane and mitochondria and polyunsaturated fatty acids(PUFAs) [3]. However, ROS dismutases, which are abundant in the skin and also currently available medications are ineffective to neutralize these most destructive radicals except Edaravone [4], of which use is strictly limited for the treatment of acute cerebral infarction patients with normal kidney and liver function. H2 may be useful in these situations because it immediately and simultaneously neutralizes both peroxynitrites and hydroxyl radicals [5] and also H2 is known to cause no significant side effects since it is produced in the human intestine as a fermentation process, although not continuously[6]. We report four cases of acute erythematous skin disease patients who were suffering from skin rash and also from associated symptoms such as severe pain and/or fever. They were treated with regular medications first and when the conventional treatments failed, then, intravenous fluids which contained H2 were added after a proper consent form was signed. However, H2 administration may not be therapeutic unless enough concentration stays at the surface layer of the skin for a sufficient period and the concentration should be higher than that of internally produced H2. Two volunteers participated in a H2 delivery study where H2 concentration in the blood and in the air at the surface of the skin was measured before, during and after H2 administration by inhalation or by intravenous fluid infusion.

## Methods

### Patients and volunteers

Before recruiting the patients and volunteers to the current study, a complete PARQ conference was given to all of the patients and their family and to volunteers. Our specific consent form, which had been approved by the Nishijima Hospital Ethics Committee and the Nishijima Hospital Pharmacists Council, was signed before the study with clear understanding of the nature of the study.

### Case history of 4 patients

#### Case 1

**Figure 1:**
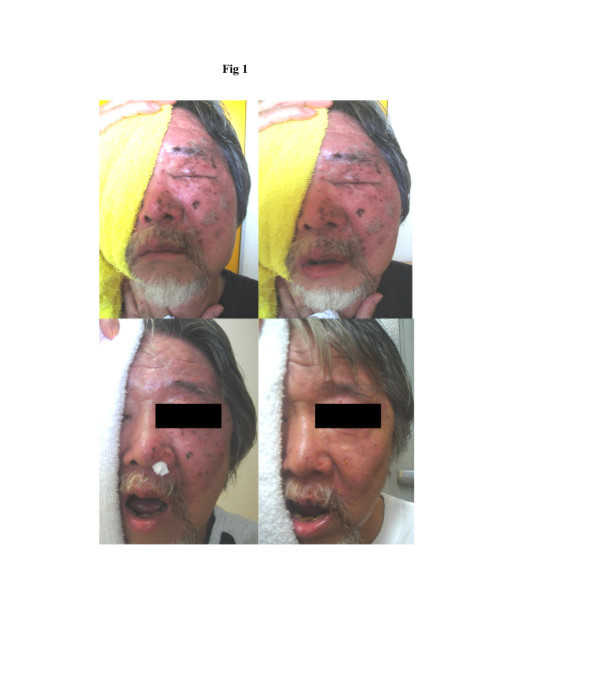
**Erythematous skin disease, Case 1.** Before the H_2_ treatment with severe erythema and edema (upper left), the patient was unable to open his left eye and the mouth except for a minimal degree with a maximal effort (upper right). Improved conditions, 3 days after the H_2_ treatment (lower left) with opening eye and mouth. The severe inflammation of the skin almost disappeared in 6 days after H_2_ treatment (lower right) and was discharged home and no return of the skin erythema noted during a follow-up period.

48 y.o. male who was in good health until 5 days prior to the admission to Nishijima Hospital when severe pain and skin rash involving his left side of the face made him to visit an emergency service where he was diagnosed as having herpes simplex infection and was treated with antivirus agents and pain medications. However, the pain increased and the left side of the face became numb. In addition, blisters in the erythematous area coalesced and formed ulcer-like appearance. The patient also noticed left ptosis and double vision and became unable to open the mouth, which made oral intake impossible. The patient was admitted to the hospital for deteriorated general condition with dehydration, severe pain and fever. On admission, the patient was found to have partial paralysis of the left 3 rd, 5th and 6th cranial nerves in addition to severe erythema with edema and small ulcers, covering the left side of the face and frontal region. The hydration treatment was initiated with 3 bags of 500 ml glucose and electrolyte solution and continued for 6 days with a decreasing dose during the hospitalization. Initially, two bags of these solutions (500 ml) had been enriched with H2. No antibiotic was given. Before the infusion therapy, the patient was unable to open his left eye and the mouth (Figure 1, upper left). The picture of Figure 1 upper right was taken after the patient was asked to open his left eye and the mouth. The patient was unable to do so, except for minimal opening of the mouth. However, 3 days after the admission and H2 infusion, the patient’s condition remarkably improved, including erythema, ulcers, pain level, opening the eye and mouth (Figure 1, lower left) and the patient became afebrile. Since cranial nerve functions recovered also and he became able to take oral soft nutrients, intravenous hydration was decreased to 2 bags of H2- enriched glucose-electrolyte solution (esuron B,200 ml/bag), daily. By the 6th hospital day, the patient was eating a regular food and his dehydration was corrected. He had no pain and the severe inflammation of the skin disappeared. The patient discharged home and no return of the skin erythema noted during a follow-up period (Figure 1, lower right).

#### Case 2

**Figure 2:**
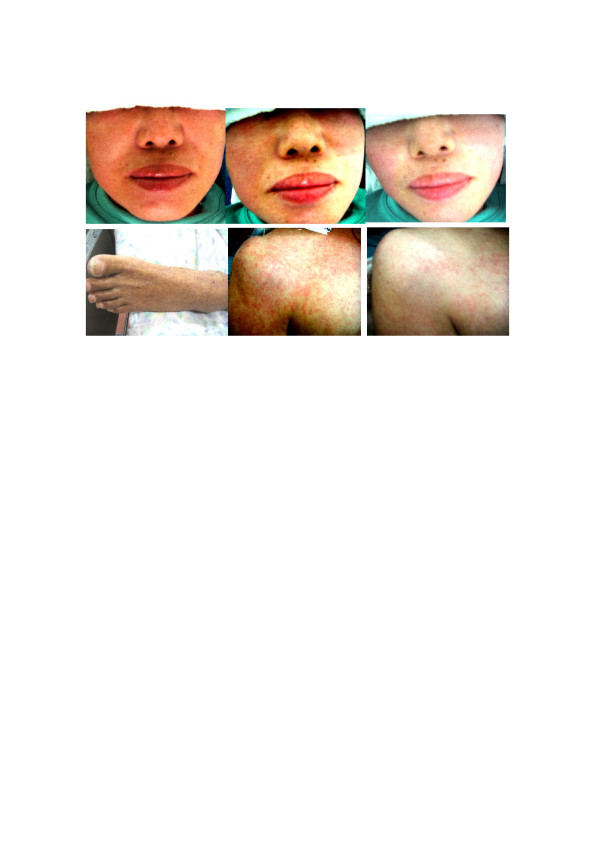
**Erythematous skin disease, Case 2, 3 and 4.** Erythematous skin lesion of the case 3 in the entire face (upper left) started improving approximately 30 min after the H2 infusion in the left side of the face first (upper-middle) and then in about one hour, the whole face improved (upper right). Severe swelling and erythema of case 2 subsided in 7 days after H2 treatment (lower left). Finer papules of case 4 started coalescing (lower middle). In 3 days after H2 treatment (lower right), significant improvement was noted and the skin lesion did not recur.

67 y.o male lapsed into coma after a large basilar artery aneurysm rupture and subarachnoid hemorrhage. After the aneurysm was surgically clipped, the patient remained comatose and developed pneumonia and cystitis, with deterioration of the liver and kidney function. After multiple medications including antibiotic and anticonvulsant, his general condition had been stabilized until 2 months after the surgery when he became febrile and developed severe skin abnormality. The abnormality consisted of erythematous papules, severe skin edema, blisters and vesicles and shedding of the skin. The Stevens-Johnson syndrome was suspected and he was transported to a general hospital with dermatology department. However, the patient was sent back with several diagnosis such as drug erythema, thrombocytopenia, possible trichophyton infection etc. and use of steroid and antifungal cream were recommended but not systemic steroid. However, application of these creams further deteriorated the skin condition despite of discontinuation of suspected drugs and finally, it was decided to use H2-enriched intravenous fluid. After a complete PARQ with the patient’s family who signed a consent, H2-enriched saline solution (500 ml) was given twice a day. Redness of the skin started fading and swelling and hardness of the skin from severe edema significantly improved in 3 days. His high fever subsided. After one week of the hydrogen treatment, the skin lesions almost disappeared (Figure 2, lower left) and general condition improved also. Although the patient remained comatose after the treatment and expired approximately 4 months after the surgery, the skin lesions did not recur.

#### Case 3

48 y.o female started feeling hot sensation in her face and developed erythema in the entire face (Figure 2, upper left) after a CT scan study with contrast enhancement for cerebral aneurysm. Drug eruption was suspected and a minophagenC solution (Minophagen Pharmaceutical Co.) which had been effective in these situations, was given intravenously. However, the erythema did not subside and the patient developed fever (38.5^○^C), headaches and nausea. As an emergency measure, two bags of a 250 ml of saline solution (Terumo Co.), which had been enriched with H2 was given. Approximately 30 min. after the infusion, the erythema started fading in the left side of the face first (Figure 2, upper middle) and then in about one hour, the whole face improved (Figure 2, upper right) and her body temperature started coming down in about one hour. At that point, the infusion stopped and the patient returned home. No recurrence of the skin rush nor fever was noted during a follow-up period.

#### Case 4

62 y.o. male had been intubated and mechanically ventilated with stable vital signs after severe subarachnoid hemorrhage from a ruptured cerebral aneurysm until 7 days after the ictus when the patient developed high fever and erythema which consisted of finer papules without fusing together. Initially, the patient was treated with local ointments with steroid but the erythema spread in the whole body and started coalescing (Figure 2, lower middle). In 3 days after H2-enriched saline solution was given twice a day intravenously, the skin lesion started fading (Figure 2, lower right) and the elevated body temperature normalized.

### Volunteers

Two volunteers who were already in Nishijima hospital with different medical conditions agreed to let the study to use H2 and their arterial access port and venous port which had been established for their medical treatment. The blood samples (1 ml at each time) were withdrawn from these ports, before, during and after H2 administration by intravenous infusion of 500 cm^3^ of saline or by inhalation of 2% H2 gas for 20 min followed by inhalation of 4% H2 gas. Both patients and their family understood perfectly that the study will not provide any benefit to them directly but possibly for the future of H2 treatment research. All the proper PARQ and signing of the consent form had been done before the initiation of the study.

### Production and administration of H2 enriched intravenous fluid and H_2_ gas

**Figure 3:**
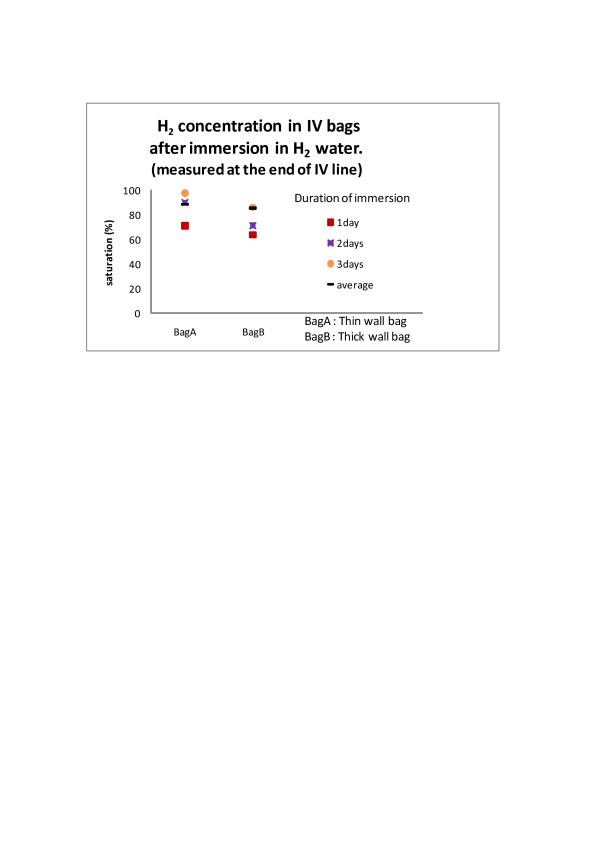
**H**_**2**_**concentration in IV bags after immersion in H**_**2**_**water.** Bag A (with soft and thin wall) vs. Bag B(with hard and thick wall). Concentration of H_2_ in BagA reached almost 93% of saturation in 48 h, while only 76% of saturation occurred in BagB in 48 h.

**Figure 4:**
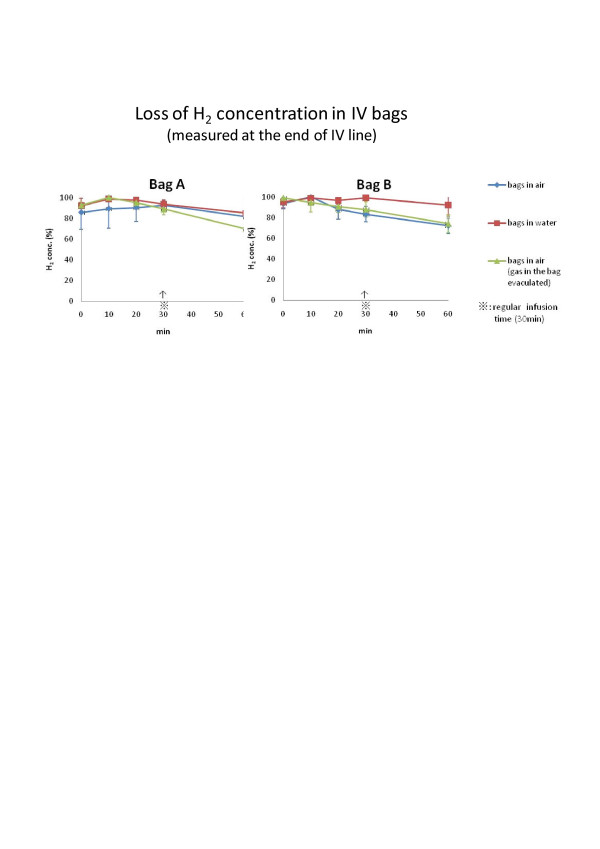
**Loss of H2 concentration in IV bags.** Bag A (with soft and thin wall) vs. Bag B(with hard and thick wall). H_2_ enriched IV bags were hanged in the air by itself (bare), or in another bag filled with water, or bare but after the gas in the bag evacuated to leave fluid only (no gas/fluid level). The bare bag still retained more than 90% of initial concentration at the end of regular infusion (30 min). Delayed desaturation occurred with the IV bag in another water-filled bag during the infusion but the set up and infusion was rather cumbersome.

H2 enriched intravenous solution was produced by simply immersing regular intravenous fluid bags in the hydrogen water tank (Miz. Co, Patent No.4486157, Patent Gazette of Japan 2010).), as has been reported elsewhere [7]. H2 gets in the bag by diffusion through the bag wall. Therefore, the bag was neither opened nor altered in anyway and this method eliminated the chance of contamination completely. Although H2 concentration in the water tank was stable at the saturation (0.8 mM), the H2 concentration in the bag varied with the duration of immersion (Figure 3) and with the material of the bag wall and by the method of infusion (Figure 4). The bag with soft and thin wall shortened the time required for the content of the bag to reach the saturation. However, the speed of loosing H2 concentration in these bags was greater than in harder and thicker wall bags, when the bag was taken out of the tank and exposed in the air for the intravenous infusion treatment. However, we have chosen the bags with softer and thinner wall because the H2 concentration, measured at the tip of the infusion catheter 30 min after the initiation of infusion, still remained approximately 90% of the original concentration and this was more convenient than using a harder and thicker wall bag with very long immersion time for saturation. By keeping the H2 enriched IV bag in another hydrogen water bag or in a bag with ice water during the infusion, the speed of desaturation decreased. However, these arrangements made the simple intravenous infusion procedure more cumbersome and complicated than searching and using a larger vein and finishing rapid intravenous infusion from a bare, soft and thin bag within 30 min. H2 gas was prepared for inhalation with an apparatus, made in our hospital by Yoji Nishijima M.D., using a H2 generator (HG200, GL Science, Tokyo, Japan), oxygen inlet and an air compressor. An appropriate concentration of H2 gas was mixed with the air and additional oxygen as needed and provided to the patient through a regular facial mask. The patients inhaled the gas by their own effort and speed but for the patients, who were on mechanical ventilation, the gas was given through the respirator.

**Figure 5:**
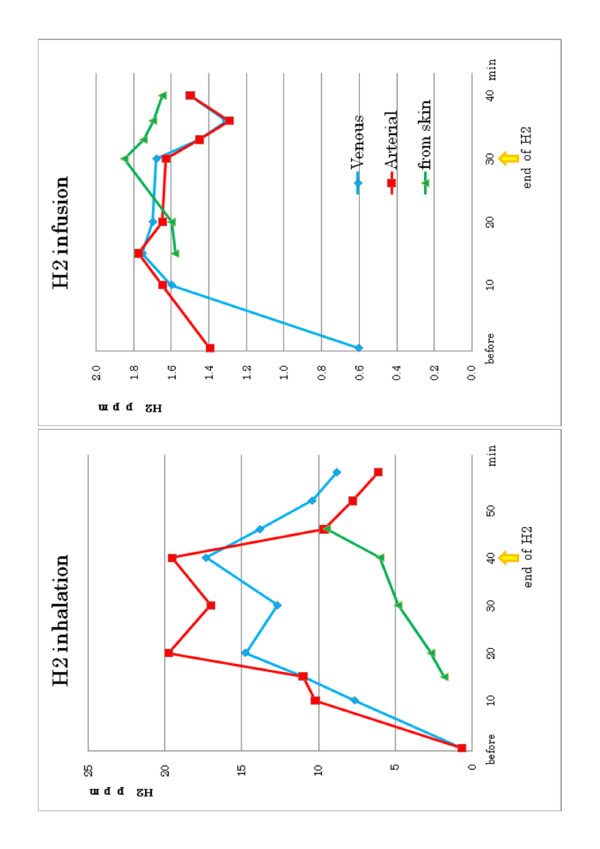
**H**_**2**_**concentration in the blood and air samples from the skin surface.** The samples were taken before, during and after H2 administration during 30 min intravenous infusion (right) and 40 min inhalation (left), for 20 min with 2% H2 gas followed by additional 20 min. with 4% H2 gas. Slower changes of H_2_ concentration are noted in the air samples from the surface of the skin as compared to the changes in the arterial and venous blood.

The production and use of the apparatus and the intravenous fluid and the gas thus produced were approved by Nishijima Hospital Pharmacists Council and were conducted upon the advice from the Council and Japanese Welfare-Labor Administration (Tokai-Hokuriku District Bureau) and Sizuoka Prefectural Administration (Pharmaceutical Affair, Regulatory Audit Section).

### H2 delivery study by measuring hydrogen concentration in the blood and in the air samples from the bag covering the leg

Ten blood samples were taken (1 cm^3^ at each time) from these arterial and venous access ports at 10, 15, 20, 30, 40, 42, 46, 52 min and 58 min after the administration of H2 and a control blood sample was taken immediately before the study.

The blood samples were immediately transferred into a small glass bottle (12 cm^3^ size) and the top was secured. The bottle was brought to a near-by gas chromatograph (TRIlyser, mBA-3000,Taiyo Co Ltd. Osaka, Japan) for the measurement of H2. For the study of H2 concentration from the skin surface, a leg of these volunteers was placed in a heavy duty, transparent plastic bag with 1 L of the air. The small glass bottles, used for the blood samples, were included in the plastic bag with the top kept open. Then, at the time of measurement during H2 administration, the top of the glass bottle was placed and secured by manipulating the glass bottle from outside of the transparent bag without opening the bag and the rest of gas in the plastic bag was suctioned away and replaced each time with 1 L of fresh air. The bottles with secured top were kept in the plastic bag until the study was completed because the plastic bag was tightly taped on the skin. Then, at the end, all the bottles with secured top were taken out of the plastic bag by removing the skin tape and were brought for the measurement of H2 concentration in the bottles by the gas chromatography. The sampling times of these air in the plastic bag were, before the study for a control and at 15, 20, 30, 40 min after starting H2 infusion and an additional sample was taken at 46 min for the inhalation study. For the H2 gas inhalation study, a 2% H2 gas was given for 20 min first and then, 4% H2 gas for additional 20 min. For H2 infusion study, 500 mL of H2 enriched saline solution was given within 30 min.

### Physiological parameters associated with H2 administration

A complete set of the physiological parameters was studied immediately before, during and after completion of H2 treatment as a routine procedure. The complete set of the parameters included 12 indices, such as body temperature(BT), blood pressure(BP), pulse rate(PR), oxygen concentration related parameters (pO2(Torr), sO2, pO2(A-a), pO2(a/A)). carbon dioxide related indices(pCO2(Torr), HCO3-act(micromole/L), and base excess related indices BE(ecf,micromole/L), BE(B,micromole/L), BB(micromole/L).

## Results

Erythema of these 4 patients and associated symptoms, such as intensive pain in the face with neurological deficits and skin ulcers (case 1), fever and edematous hardening of the entire body, particularly in the extremities with skin ulcers (Case 2), rather mild but with acute fever and nausea and headache (case 3), mild but worsening and spreading skin lesions with fever (case 4), all improved significantly after the H2 treatment and did not recur.

The H2 delivery study of two volunteers showed that the concentration of H2 in the blood rapidly increased with H2 inhalation and slowly decreased with cessation of H2, particularly in the venous blood. However, H2 concentration of the air samples in the plastic bag covering a leg showed much slower changes and continued to increase even after H2 inhalation was discontinued, at least during the time of sample collection (Figure 5). The blood level of H2 was significantly higher when H2 was given by inhalation as compared to via intravenous route.

Administration of H2 did not change physiological parameters and did not cause significant deterioration of the blood chemistry, although some of these patients already had severe abnormalities before the H2 treatment such as thrombocytopenia of case 2 (Table 1).

**Table 1:** Duration of H2 treatment and lab studies of 4 patients

	**chemistry**					**hematology**		
	**GOT**	**GPT**	**γ-GPT**	**T-BIL**	**CRP**	**WBC**	RBCx10^4^	Plateletx10^4^
**Case 1 : 6 days**								
At the beginning	23	58	96	0.7	1.51	7800	462	32.2
4 days after	16	27	66	0.4	1.89	7800	430	36.8
9 days after (3 days after resumption)	20	22	63			10600	431	52.8
22 days after, (16 days After resumption)	17	12	45		0.19	8000	440	51.6
	**chemistry**					**hematology**		
	**GOT**	**GPT**	**γ-GPT**	**T-BIL**	**CRP**	**WBC**	**RBCx10**^**4**^	**Plateletx10**^**4**^
**Case 2:19 days**								
At the beginning	33	58	253	0.6	8.27	10200	367	29.1
7 days after	20	57	244		8.71	18800	297	20.1
10 days after	27	59	233		9.35	37000	348	3.8
2 weeks after	29	46	210	1.9	6.53	11900	288	1.9
18 days after	19	35	152	1.6	5.81	11200	258	4.6
3 weeks after	15	22	79		8.64	17700	310	24.2
	**chemistry**					**hematology**		
	**GOT**	**GPT**	**γ-GPT**	**T-BIL**	**CRP**	**WBC**	**RBCx10**^**4**^	**Plateletx10**^**4**^
**Case 3 : 2hrs**								
At the beginning	22	25	28			4500	432	13.6
45 days after (45 days after completion)	19	20	25	0.4	0.24	4500	439	15.5
	**chemistry**					**hematology**		
	**GOT**	**GPT**	**γ-GPT**	**T-BIL**	**CRP**	**WBC**	**RBC (万)**	**Plateletx10**^**4**^
**Case 4 : 3 days**								
At the begining	25	35	342		1.31	18000	396	30.6
2 days after	34	36	286		0.65	21900	381	26
5 days after (2 days after completion)	60	117	300		1.91	13200	409	11.7

Administration of H2 did not change physiological parameters and did not cause significant deterioration of the blood chemistry such as for liver and kidney function, although some of these patients already had severe abnormalities before the H2 treatment such as thrombocytopenia of case 2.

## Discussion

In neurosurgery, administration of multiple medications for the treatment of depressed level of consciousness, elevated intracranial pressure, pneumonia, abnormal diuretic hormone etc. are very common and immediate discontinuation of these regimens due to suspected drug eruption is rather difficult, if not impossible. It is well known that in acute erythematous diseases of the skin, ROS (reactive oxygen species) produced by migrated inflammatory cells and others destroy the cell membrane and aggravate the skin disease [1,2]. Peroxynitrites and hydroxyl- radicals are most potent and cannot be eliminated by clinically available medications except only by edaravone [4]. However, edaravone is approved only for acute stage of cerebral infarction in Japan and the high cost of the medication and possibility of side effects make frequent and prolong use difficult. In addition, edaravone becomes a radical by itself after neutralizing the surrounding radicals which needs to be neutralized by other materials. On the other hand, H2 inactivates peroxynitrites and hydroxyl radicals directly and immediately without any risk of side effects and also H2 can be produced easily with low cost. However, H2 until this time has been provided in drinking water or as a gas and could not be administered when oral intake or inhalation is prohibited from a medical condition.

Recently, a new technique allowed hydrogen to be dissolved safely [7] in the bag of intravenous fluid solution by simply immersing the bag in a H2 water producing container. H2 gets in the bag by simple diffusion and therefore, no need for opening the bag or any alteration of the bag, which eliminates the risk of contamination and infection. All of our 4 cases in this report were unable to drink or swallow and it was thought that H2 could be given only by intravenous route. The safety monitoring with physiological parameters and laboratory studies showed no ill effects on those multiple indices and organ function such as kidney and liver function, by this method of H2 administration (Table 1). Even in the case 2 with thrombocytopenia, no other hematological worsening was noted. Clinical symptoms of the skin diseases of all four patients improved rather rapidly and significantly. Therefore, it may be reasonable to assume that H2 infusion in these situations was quite safe and effective. However, it is still possible that the skin condition improved for reasons unrelated to H2 administration since the pattern of improvement was not uniform and a clear dose response relation could not be obtained. The clinical effectiveness of hydrogen is usually explained by its specific scavenger ability against hydroxyradicals and peroxynitrites. The first line of the defense in the skin is keratinocytes which are located in the outermost layer of the skin and known to produce a large quantity of ROS (reactive oxygen species), primarily for antimicrobial effects and reduction of microbial virulence factors at high level [8]. Although many ROS scavengers and dismutases are present in the skin where severe oxidation continues all the time, it is essential to maintain a low level ROS for regulatory purposes [9]. These residual ROS and ROS overflow from overproduction can be the source of peroxynitrites. In addition, intracellular peroxynitrites are generated by the Nox 1 ((NADPH oxidase 1) -derived ROS and intracellular NO (nitric oxide), since Nox1 is localized in the nucleus of keratinocytes [10]. Peroxynitrites activates p38MAPK pathways, which are related to production of inflammatory cytokines, such as TNF-α and IL-1, and many others [11]. The control of these intracellular processes by diffusible H2 is important and thus H2 may have reduced the level of inflammation seen in erythematous skin disease. In the present H2 concentration study, the blood concentration was much lower when H2 was given via intravenous route as compared to inhalation. However, in the both modes of H2 administration, the arterial blood concentration was elevated first and then venous blood but the H2 concentration in the air around the skin increased very slowly and then continued to increase even after H2 administration by inhalation stopped. These findings may imply that the slowness is related to the slow blood flow compartment [12] and/or permeability barrier of lipid-water layers of the skin tissue itself [13] and also that the skin may function as a reservoir for a prolonged release of H2. The slow but prolonged release of H2 directly from the skin is most advantageous as a therapeutic regimen of the skin diseases, since the erythematous skin disease involves outermost layer of the skin which is exposed to continuous oxidation and possesses rather scarce blood vessels. If the production of ROS is continuous, the delivery of H2 needs to be longer. The skin with a slower desaturation compartment provides better opportunity for utilization of intravenous infusion as a useful route for H2 administration, since the slower compartment may accumulate more H2 with prolonged administration rather than higher concentration with relatively shorter administration, such as by inhalation. Intravenous administration of H2 was more convenient and consistent from our experience, since it was usually difficult to keep a facial mask on conscious but neurologically compromised patients for a prolonged time for H2 inhalation. The limitations of our study include small number of patients and volunteers. Our conclusions from the results of this study need to be confirmed by a study with more patients and volunteers. Possibility of presence of a slow H2 desaturation compartment in the skin needs to be examined with a much longer study time. Because this study was done on volunteers, our study time was limited and too short for the analysis of the slow releasing compartment. In addition, no tissue biopsy was done from the skin. Therefore, no final diagnosis of the skin disease was available. Future studies will clarify those issues and provide a best selection of H2 delivery method based upon the nature of the disease and the condition of the patient.

In summary, erythema of these 4 patients and associated symptoms, such as intensive pain in the face with neurological deficits and skin ulcers, fever and edematous hardening of the entire body, rather mild but with acute fever and nausea and headache, mild but worsening and spreading skin lesions with red rush all improved significantly after the H2 treatment and did not recur. The H2 delivery study of two volunteers showed that the concentration of H2 in the blood rapidly increased with H2 inhalation and slowly decreased with cessation of H2, particularly in the venous blood. However, H2 concentration of the air samples in the plastic bag covering a leg showed much slower changes and continued to increase even after H2 inhalation was discontinued, at least during the time of sample collection.

## Competing interests

The authors declare that they have no competing interests and were not compensated at all by any pharmaceutical and biotechnology company to contribute this article to the peer-reviewed scientific literature.

## Authors’ contributions

The authors equally contributed to the production of this article.

## References

[B1] Robert C Kupper TS Inflammatory skin diseases, T cells, and immune surveillance N Eng J Med 1999 341 1817 1828 10.1056/NEJM199912093412407 10588968

[B2] Ely JW Stone MS The generalized rash: Part1. Differential diagnosis. Am Fm Physician 2010 81 726 734 20229971

[B3] Trenam CW Blake DR Morris CJ Skin inflammation: Reactive oxygen species and the role of iron J Invest Dermatol 1992 99 675 682 10.1111/1523-1747.ep12613740 1469283

[B4] Zhang N Komine-Kobayashi M Tanaka R Liu M Mizuno Y Urabe T Edaravone reduces early accumulation of oxidative products and sequential inflammatory responses after transient focal ischemia in mice brain Stroke 2005 36 2220 2225 10.1161/01.STR.0000182241.07096.06 16166574

[B5] Ohsawa I Ishikawa M Takahashi K Watanabe M Nishimaki K Yamagata K Katsura K Katayama Y Asoh S Ohta S Hydrogen acts as a therapeutic antioxidant by selectively reducing cytotoxic oxygen radicals Nat Med 2007 13 688 694 10.1038/nm1577 17486089

[B6] Levitt MD Production and excretion of hydrogen gas in man N Eng J Med 1969 281 122 127 10.1056/NEJM196907172810303 5790483

[B7] Ono H Nishijima Y Adachi N Tachibana S Chitoku S Mukaihara S Sakamoto M Kudo Y Nakazawa J Kaneko K Nawashiro H Improved brain MRI indices in the acute brain stem infarct sites treated with hydroxyl radical scavengers, Edaravone and hydrogen as compared to Edaravone alone. A non-randamized study. Medical Gas Research 2011 1 12 20 10.1186/2045-9912-1-12 22146068 PMC3231971

[B8] Grange PA Chereau C Raingeaud J Nicco C Weill B Dupin N Batteux F Production of superoxide anions by keratinocytes initiates P. acnes-induced inflammation of the skin PLoS Pathogens 2009 5 1 14 10.1371/journal.ppat.1000527 PMC2709429 19629174

[B9] Bedard K Krause K-H The NOX family of ROS-generating NADPH oxidases: Physiology and pathophysiology Physiol Rev 2007 87 245 313 10.1152/physrev.00044.2005 17237347

[B10] Chamulitrat W Schmidt R Tomakidi P Stremmel W Chunglok W Kawahara T Rokutan K Association of gp91phox homolog Nox1 with anchorage-independent growth and MAP kinase activation of transformed human keratinocytes Oncogene 2003 22 6045 6053 10.1038/sj.onc.1206654 12955083

[B11] Huang C-S Kawamura T Toyoda Y Nakao A Recent advances in hydrogen research as a therapeutic medical gas Free radical Reesearh 2010 44 971 982 10.3109/10715762.2010.500328 20815764

[B12] Aukland K Bower BF Berliner RW Measurement of localblood flow with hydrogen gas Circ Res 1964 14 164 187 10.1161/01.RES.14.2.164 14118761

[B13] Treherne JE The permeability of skin to some nonelectrolytes J Phsiol 1956 133 171 180 10.1113/jphysiol.1956.sp005575 PMC1359146 13346643

